# Morphological Analysis of Nose in Patients of Tessier No. 0 Cleft With a Bifid Nose in China

**DOI:** 10.3389/fped.2021.768176

**Published:** 2021-11-29

**Authors:** Xin Wang, Huan Wang, Jianjun You, Ruobing Zheng, Yihao Xu, Xulong Zhang, Junsheng Guo, Fei Fan

**Affiliations:** Department of Rhinoplasty, Plastic Surgery Hospital, Chinese Academy of Medical Sciences and Peking Union Medical College, Beijing, China

**Keywords:** bifid nose, morphological analysis, Tessier no. 0 cleft, facial cleft, anthropometric measurements

## Abstract

**Objective:** Facial cleft involves complex malformations. No study assessed the facial deformity of Tessier No. 0 cleft with a bifid nose. Thus, we used anthropometric measurements to access the nose in patients.

**Methods:** A total of 24 bifid nose deformities underwent surgery at our institution between 2010 and 2019. Standardized photographs were taken preoperatively and postoperatively. Landmarks were identified on these images; measurements for nasal analysis were performed and compared with the established Chinese norms. Surgical method differences were also analyzed.

**Results:** The median follow-up time was 2.51 years. Postoperatively, there is a significant difference in comparison with preoperative in the nasal index, medial canthus and nose width index, nasolabial angle, nasofacial angle, ala length and nasal bridge length index, nasal tip protrusion and nasal width index, and nasal width and ala length index. Furthermore, the medial canthus and nose width index, and nasal width and ala length index were significantly larger in ordinary people, while ala length and nasal bridge length index and nasal tip protrusion and nasal width index were smaller. After surgery, most angles and index were standard except the nasolabial angle in the females, and ala length and nasal bridge length index in the males. Moreover, as for the group of costal cartilage transplantation, most index and angles have improved after surgery including nasolabial angle, nasofacial angle, ala length and nasal bridge length index, nasal tip protrusion and nasal width index, and nasal width and ala length index. However, only nasal tip protrusion and nasal width index, columella length and nasal tip protrusion index, and nasal width and ala length index in the silicone prosthesis group implantation has significance. Costal cartilage transplantation can also better improve ala length and nasal bridge length index than the silicone prosthesis implantation.

**Conclusion:** Most defects can be repaired with surgery, but the outcome has a lack of evaluation. Thus, anthropometric assessment can serve as a material for nasal and reconstructive surgery.

## Introduction

Craniofacial clefts, also called facial clefts, are rare congenital malformations, usually involving multiple facial parts and aesthetic units. In 1976, Tessier classified the facial clefts based on his personal experience into a number from 0 to 14 (1). Though the exact incidence is unknown, the new birth rate is approximately ranged from 1.4 to 4.9 per 100,000 births (1–3). Among them, facial cleft involving nasal subunits such as nasal dorsum, alar, tip, and columella is usually called a bifid nose, which is regarded as the most common craniofacial cleft and corresponds to no. 0 of the Tessier classification (2–4). The clinical manifestations are variable, and the severity is different. The patients may present a nasal dorsum that is collapsed, and the flat nasal tip is faintly grooved or deeply furrowed, or alar cartilages are split. The nose usually looks very short with or without orbital hypertelorism.

Although the incidence rate of bifid is low [about 0.0008% (5)], the development of the face has been seriously affected by its unique malformation and various clinical manifestations. The nose is the central feature of the face, which has a profound influence on facial aesthetics. Thus, the bifid nose will have a significant impact on the physical and mental health of the patients. Rhinoplasty is an essential part of the treatment of facial cleft. Compared with ordinary people, the operation is more complicated. Surgical correction is challenging due to rare cases and complex current deformities. Thus, the surgical methods have not been unified, and a quality assessment of surgical outcomes is required.

In recent years, scholars worldwide have done a lot of anthropometric studies on the normal face and nose, and even on the secondary deformities in cleft lip and palate patients in plastic and maxillofacial surgery (6, 7). Thus, exact anthropometric measurements are necessary to detect surgical shortcomings and to focus further efforts on them, but domestic and foreign scholars have not studied the facial features of Tessier no. 0 cleft with a bifid nose. Our study objectively analyzed the deformities of the bifid nose before and after surgery, and found the defects between the patients and ordinary people. We hope this measurement could show that our surgical methods could improve the nasal defect of patients and obtain good aesthetic effects in a way of morphological analysis.

## Methods

### Patients

Through the period between 2010 and 2019, a total of 24 patients, who were diagnosed with Tessier no. 0 cleft with bifid nose and received surgery in our hospital, were included in this retrospective study. Ethics approval was granted in our department, and we confirmed that all methods were performed following the relevant guidelines and regulations. We collected the data of each patient, including sex, age at surgery, and operation method. Baseline data are presented in Table 1.

**Table 1:** Patients' characteristics.

**Patient's characteristics**	**Number**
Number of patients	24
**Sex**	
Male (frequency)	13 (54.17%)
Female (frequency)	11 (45.83%)
Age at surgery (median)	5.62 ± 4.82
Age at the last follow up (median)	8.13 ± 5.64
**Operation method**	
Nose reconstruction (frequency)	14 (58.33%)
Silicone augmentation rhinoplasty (frequency)	10 (41.67%)

### Photogrammetric Measurements

Digital photographs were taken from frontal, lateral, and submental views before and after surgery, which satisfied the criteria mentioned by Kohout et al. (8). During the shooting, the patient sat in a natural resting posture, relaxed their nose and facial muscles, looked straight ahead, closed their lips slightly, and their hair was tied up to reveal the auricle. In the frontal, lateral view, the plane from the upper edge of the external auditory canal to the lower edge of the orbit is parallel to the ground. In the opinion regarding the submental, the line between the upper eyelids and the tip of the nose should be in a horizontal line.

**Figure 1:**
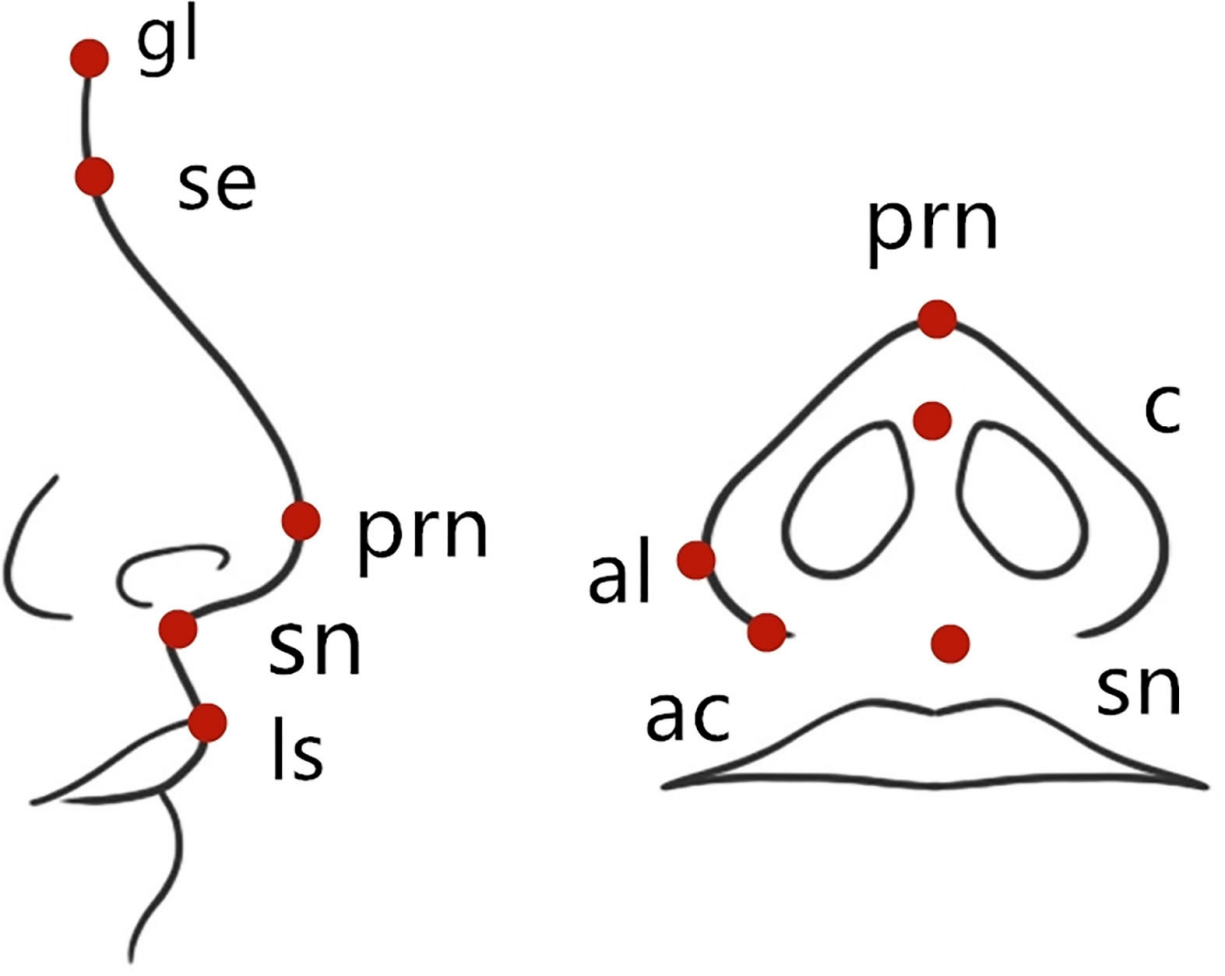
A schematic representation to show the standard anthropometric landmarks.

Anthropometric proportions are mentioned by Gewalli et al. (9) and Holmstrm and Gewalli (10), and research reports on facial cleft. The landmarks are shown in Figure 1. Significant landmarks included endocanthion (en), alare (al), sellion (se), subnasale (sn), glsbella (gl), pronasale (prn), alar crest (ac), columella (c), and labiale superius (ls). The measured angle and index included the nose index (al-al/se-sn, the ratio of nose width to height), inner canthus and nose width index (en-en/al-al; the ratio of inner canthus distance to nose width), nasolabial angle (c-sn-ls), nasofrontal angle (gl-se-prn), nasofacial angle (the angle between the line from the point of “se” to the point of “prn,” and the vertical line of the nasal root), ala length and nasal bridge length index (prn-ac/se-prn), nasal tip protrusion and nasal width index (prn-sn/al-al), columella length and nasal tip protrusion index (c-sn/prn-sn), and nasal width and ala length index (al-al/ac-prn).

Besides, the nasal and facial data of ordinary Chinese youth were from the PubMed search engine using the following keywords: “Chinese” or “Nose” or “Nasal” or “Facial” or “Morphological Analysis” or “Photogrammetric” or “Anthropometric” or “Measurement.” The search was restricted to English language publications without date limitations. The literature containing healthy Chinese data of nose measurement was included.

### Follow-Up

All patients were followed as long as we can. For patients who completed multiple surgeries, only the recently taken photos were used for data analysis.

### Surgical Techniques

All patients were administered general anesthesia. For patients with more nasal subunit defects, we used the forehead expansion flap and costal cartilage transplantation. The first stage of surgery is the placement of the forehead dilator under the galea aponeurosis, injecting normal saline into the expander to 600–800 ml gradually. The second stage of the operation is to take the costal cartilage, transfer the forehead flap, and reconstruct the whole nose. According to the shape of the nose, a variety of grafts, such as nasal dorsal, nasal tip, nasal columella, and nasal alar margin, were carved and sutured. The expanded forehead flap is usually pedicled with supratrochlear vessels or supraorbital vessels. The rotation point is determined, the flap is designed retrogradely, and the flap is placed in the nasal defect. The incision was intended on the nasal columella. The skin of the nasal dorsum and nasal columella were used to form flaps, respectively, which were turned inward to form bilateral nostril lining. Finally, the grafts were placed in the nasal cavity. The third stage is flap pedicle division. Then it is mainly the revision of the local flap of nasal reconstruction.

For patients with mild nasal deformity, we used silicone prosthesis implantation and local flaps. According to the defect situation, we used a Z-shaped flap, bilobed flap, or trilobal flap to repair the alar defect and improve the shape of the nostril. At the same time, we cut through the medial margin of the nostril and put the L-type silicone prosthesis into the nasal dorsal superficial fascia.

### Statistical Analysis

Analysis was performed using MB-Ruler 4.0 by Markus Bader. Measurements were taken independently by two examiners (XW and WFD). All statistical analyses were performed using statistical software (SPSS Version 22.0). Thus, we can get the clinical characteristics of Tessier no. 0 cleft with a bifid nose. Measurements were compared with that of published literature. Summary statistics were provided as percentages. The paired Student *t*-test was used for the preoperative and postoperative morphometric analysis of the nose between men and women, and between different surgery methods. Data were represented as means ± standard error of the mean. A *p*-value < 0.05 was considered statistically significant.

## Results

**Figure 2:**
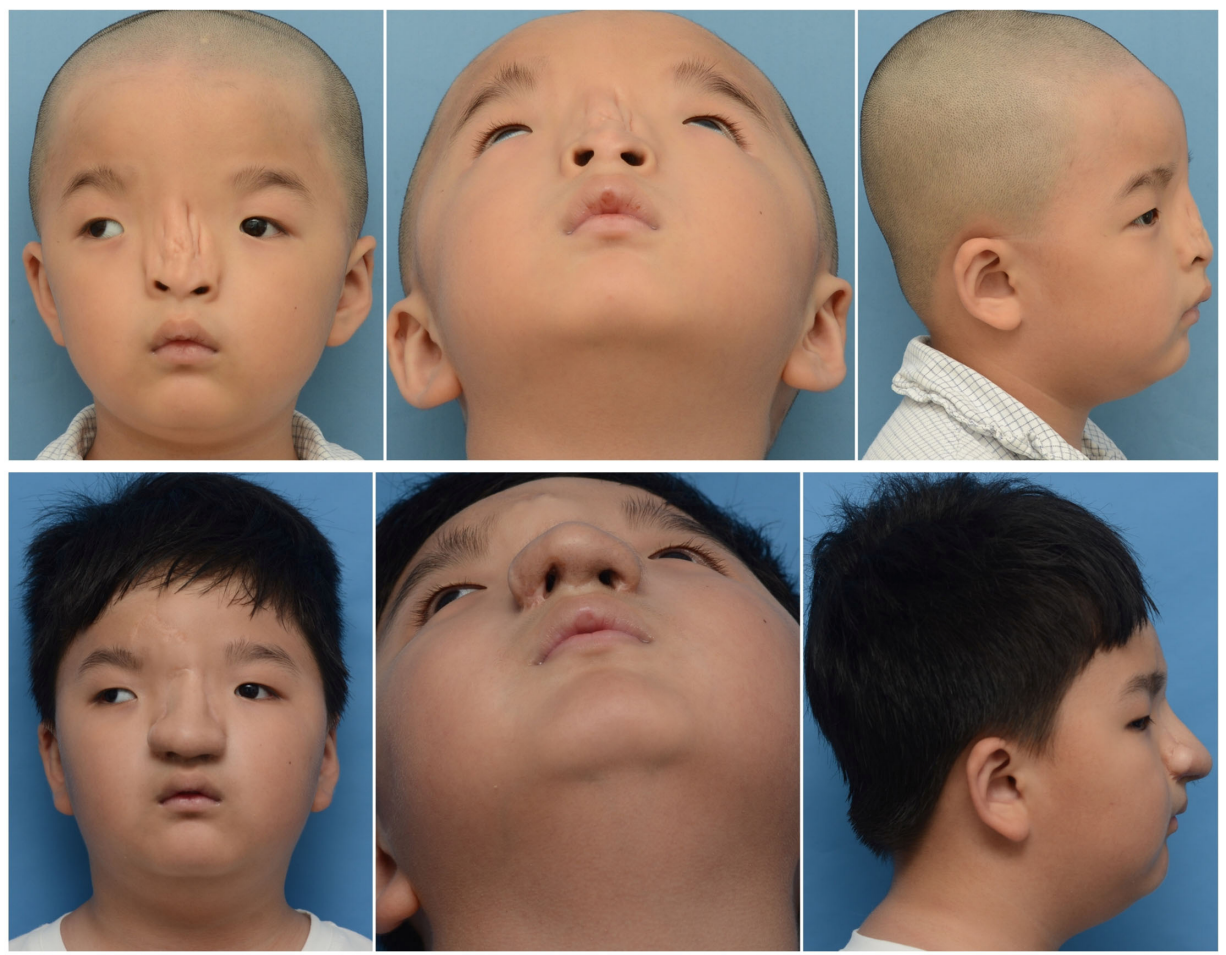
A 6-year-old boy has flat and broad nasal dorsum accompanied by a more deeply furrowed with extremely low and flat nasal tip. Due to the deep groove of the nasal dorsum, and the shape of the nose could not be improved by flap transfer, he was treated with forehead expansion flap and costal cartilage transplantation. The above three pictures were preoperative photos. The following pictures were the follow-up photos after six operations.

**Figure 3:**
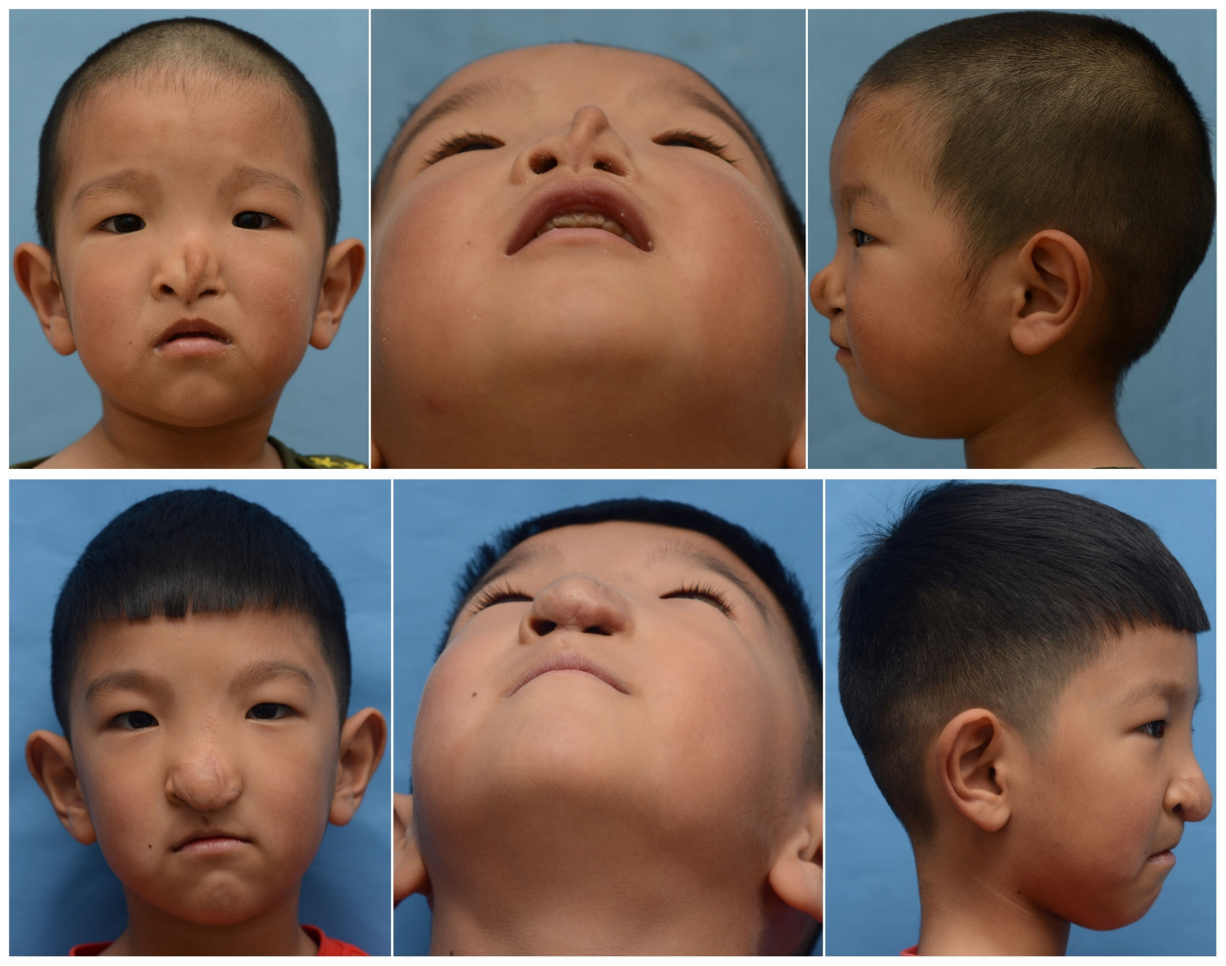
A 4-year-old boy, with collapsed and flat nasal dorsum, underwent local flaps and silicone prosthesis implantation. The above three pictures were preoperative photos. The following pictures were the follow-up photos after two operations.

A total of 24 patients were treated in our center with a male significant predominance (M/F = 1.18) between 2010 and 2019. The median age of the patients at surgery in our hospital was 5.62 years (range 1–18 years). The median follow-up time was 2.51 years (range 1–4 years). Moreover, 10 people were treated with local flaps and silicone prosthesis implantation for the operation, while 14 people were treated with forehead expansion flap and costal cartilage transplantation. Table 1 shows the summary of the characteristics of the patients. We also chose one patient for each of the two surgical methods as an example (Figures 2, 3).

All 24 patients had satisfactory results after the operation. We also used photogrammetric measurements to evaluate the changes before and after the operation. Postoperatively, there is a significant difference in comparison with preoperative in nasal index, medial canthus and nose width index, nasolabial angle, nasofacial angle, ala length and nasal bridge length index, nasal tip protrusion and nasal width index, and nasal width and ala length index (*p* < 0.001), which are summarized in Table 2.

**Table 2:** Comparison of angle and index measurements before and after operation.

	**Preoperative**	**Postoperative**	***P*** -value^*^
Nasal index	0.93 ± 0.17	0.83 ± 0.08	0.016
Medial canthus & nose width index	1.30 ± 0.21	1.19 ± 0.15	0.043
Nasolabial angle	107.62°±19.12°	84.54°±13.86°	<0.001
Nasofrontal angle	148.92°±12.12°	142.33°±12.16°	0.072
Nasofacial angle	25.28°±6.14°	32.51°±5.42°	<0.001
Ala length & nasal bridge length index	0.38 ± 0.09	0.47 ± 0.12	0.003
Nasal tip protrusion & Nasal width index	0.29 ± 0.07	0.50 ± 0.10	<0.001
Columella length & Nasal tip protrusion index	0.57 ± 0.10	0.55 ± 0.62	0.892
Nasal width & ala length index	1.90 ± 0.23	1.45 ± 0.33	<0.001

*Values are means ± standard deviations*.

* *Indicates the two pair samples Student t-test*.

To further explore the facial features in Tessier no. 0 cleft with a bifid nose, we used the search filter. Eleven articles were found through PubMed, which conformed to our criteria, including the chosen index and angles in ordinary Chinese people after careful reading of the complete manuscript (11–22), summarized in Table 3. Through the summary and analysis of the data of each literature, we can get the average data of the Chinese people. When comparing with the ordinary people, we could find that there was a statistical difference in the medial canthus and nose width index, nasofrontal angle, ala length and nasal bridge length index, nasal tip protrusion and nasal width index, and nasal width and ala length index in the males, and medial canthus and nose width index, nasofacial angle, ala length and nasal bridge length index, nasal tip protrusion and nasal width index, and nasal width and ala length index in the females, respectively. It is noteworthy that most items were not statistically significant after surgery, except the medial canthus and nose width index, and ala length and nasal bridge length index in the males, and medial canthus and nose width index, and nasolabial angle in the females. All of the details are summarized in Table 4.

**Table 3:** Summarize the nasal angles in the present studies.

**References**	**Sex**	**Nasal Index**	**Medial canthus & nose width index**	**Nasolabial angle**	**Nasofrontal angle**	**Nasofacial angle**	**Ala length & nasal bridge length index**	**Nasal tip protrusion & Nasal width Index**	**Columella length & Nasal tip protrusion Index**	**Nasal width & ala length Index**
Dong et al. (11)	M	0.88	0.86	104.30	138.19	—	—	0.38	—	—
	F	0.80	0.94	103.42	144.04	—	—	0.37	—	—
Farkas et al. (12)	M	0.73	0.96	—	—	—	—	—	—	—
	F	0.72	0.97	—	—	—	—	—	—	—
Aung et al. (13)	M	0.85	—	86.9	134.5	—	0.67	0.41	—	1.27
	F	0.84	—	88.5	135.6	—	0.63	0.41	—	1.33
Leong and White (15)	M	0.85	—	86.2	129.3	34.70	—	—	—	—
	F	0.89	—	87.6	136.6	33.70	—	—	—	—
Zhang et al. (16)	M	0.78	0.94	—	—	34.33	—	0.46	—	—
	F	0.75	1.01	—	—	34.68	—	0.47	—	—
Rhee et al. (17)	M	—	—	—	—	—	—	—	—	—
	F	—	—	113.51	—	—	—	—	—	—
Jayaratne et al. (18)	M	0.85	—	102.14	141.01	—	0.67	0.49	0.69	1.27
	F	0.78	—	105.60	143.51	—	0.64	0.48	0.67	1.22
Dong et al. (19)	M	—	—	104.3	138.19	—		—	—	—
	F	—	—	103.42	144.04	—		—	—	—
Liu et al. (20)	M	0.89	1.00	117.68	—	—		—	—	—
	F	0.88	1.05	119.04	—	—		—	—	—
Li et al. (21)	M	0.75	0.99	100.99	132.6	—		0.53	0.51	1.28
	F	0.74	0.99	98.97	138.7	—		0.54	0.53	1.23
He et al. (22)	M	0.65	0.95	98.5	138.15	—	0.62	0.45	0.46	1.21
	F	0.60	1.04	100.05	147.71		0.57	0.47	0.48	1.21
Mean value	M	0.82 ± 0.07	0.95 ± 0.05	100.13 ± 9.48	135.99 ± 3.74	34.52 ± 0.19	0.65 ± 0.02	0.45 ± 0.05	0.55 ± 0.10	1.26 ± 0.03
	F	0.78 ± 0.08	1.00 ± 0.04	102.23 ± 9.67	141.46 ± 4.18	34.19 ± 0.49	0.61 ± 0.03	0.46 ± 0.05	0.56 ± 0.08	1.25 ± 0.05

**Table 4:** Comparison of nasal angles and index between females and males and norm respectively.

	**Male**				**Female**			
	**Preoperative value**	**Postoperative value**	***P*** -value (pre vs. normal)^*^	***P*** -value (post vs. normal)^*^	**Preoperative value**	**Postoperative value**	***P*** -value (pre vs. normal)^*^	***P*** -value (post vs. normal)^*^
Nasal Index	0.90 ± 0.18	0.81 ± 0.08	0.677	0.086	0.96 ± 0.16	0.85 ± 0.06	0.999	0.498
Medial canthus & nose width index	1.32 ± 0.24	1.18 ± 0.16	<0.001	0.011	1.28 ± 0.16	1.20 ± 0.14	0.001	0.002
Nasolabial angle	106.32 ± 18.34	88.14 ± 13.40	0.080	0.260	109.65°±19.89°	80.29 ± 13.17	0.152	0.004
Nasofrontal angle	150.4 ± 13.49	144.28 ± 13.21	0.006	0.100	147.18°±9.99°	140.03 ± 10.31	0.135	0.687
Nasofacial angle	24.17 ± 7.23	30.13 ± 5.70	0.067	0.417	26.82°±4.08°	35.32 ± 3.33	0.028	0.147
Ala length & nasal bridge length index	0.38 ± 0.09	0.48 ± 0.10	<0.001	0.006	0.36 ± 0.09	0.45 ± 0.14	0.026	0.281
Nasal tip protrusion & Nasal width Index	0.32 ± 0.07	0.49 ± 0.10	0.002	0.547	0.27 ± 0.06	0.50 ± 0.09	*P* < 0.001	0.683
Columella length & Nasal tip protrusion Index	0.56 ± 0.08	0.67 ± 0.82	0.570	0.781	0.58 ± 0.11	0.41 ± 0.10	0.224	0.12
Nasal width & ala length Index	1.88 ± 0.23	1.47 ± 0.27	*P* < 0.001	0.191	1.92 ± 0.24	1.41 ± 0.38	<0.001	0.514

*Values are means ± standard deviations*.

* *Indicates the two pair samples Student t-test*.

Tessier no. 0 cleft with bifid nose usually involves multiple nasal subunits, so surgical strategies will vary with the severity of the deformity, but the primary repair is in the nasal dorsum. There are two main ways to operate, and one is the forehead expansion flap and costal cartilage transplantation, another is the local flaps and silicone prosthesis implantation. Therefore, we compared the effects of the two operations and summarized them in Table 5. As for the group of costal cartilage transplantation, most index and angles have improved after surgery, including the nasolabial angle, nasofacial angle, ala length and nasal bridge length index, nasal tip protrusion and nasal width index, and nasal width and ala length index. However, the group of silicone prosthesis implantation was not prominent; only the nasal tip protrusion and nasal width index, columella length and nasal tip protrusion index, and nasal width and ala length index have significance. Moreover, we also studied the difference in the degree of improvement between the two methods, which found that costal cartilage transplantation can better improve ala length and nasal bridge length index than the silicone prosthesis implantation, but there was no significant difference between the two methods for the other items (Table 6).

**Table 5:** Comparison of nasal angles and index in different surgical methods.

	**Forehead expansion flap + Costal cartilage transplantation**			**Local flaps + Silicone prosthesis implantation**		
	**Preoperative**	**Postoperative**	***P*** -value^*^	**Preoperative**	**Postoperative**	***P*** -value^*^
Nasal Index	0.94 ± 0.18	0.83 ± 0.09	0.068	0.90 ± 0.15	0.82 ± 0.06	0.128
Medial canthus & nose width index	1.28 ± 0.21	1.19 ± 0.15	0.185	1.32 ± 0.21	1.19 ± 0.15	0.142
Nasolabial angle	106.39°±16.86°	78.45°±13.83°	<0.001	109.34°±21.78°	93.07°±8.25°	0.059
Nasofrontal angle	149.85°±11.62°	141.42°±10.91°	0.068	147.62°±12.66°	143.61°±13.61°	0.526
Nasofacial angle	24.59°±6.01°	33.09°±5.35°	0.001	26.50°±6.14°	31.70°±5.42°	0.073
Ala length & nasal bridge length index	0.35 ± 0.09	0.51 ± 0.10	<0.001	0.40 ± 0.09	0.41 ± 0.13	0.835
Nasal tip protrusion & Nasal width Index	0.31 ± 0.07	0.50 ± 0.09	<0.001	0.28 ± 0.06	0.50 ± 0.11	<0.001
Columella length & Nasal tip protrusion Index	0.55 ± 0.09	0.67 ± 0.79	0.583	0.60 ± 0.10	0.39 ± 0.10	<0.001
Nasal width & ala length Index	1.86 ± 0.26	1.48 ± 0.22	0.001	1.96 ± 0.16	1.40 ± 0.43	0.002

*Values are means ± standard deviations*.

* *Indicates the two pair samples Student t-test*.

**Table 6:** Comparison of nasal angles and index of improvement value between the two groups.

	**Forehead expansion flap + Costal cartilage transplantation**	**Local flaps + Silicone prosthesis implantation**	***P*** -value^*^
Nasal Index	−0.11 ± 0.19	−0.08 ± 0.16	0.738
Medial canthus & nose width index	−0.10 ± 0.17	−0.13 ± 0.16	0.593
Nasolabial angle	−27.94°±17.55°	−16.26°±22.70°	0.188
Nasofrontal angle	−8.43°±15.69°	−4.01°±18.93°	0.556
Nasofacial angle	8.50°±6.53°	5.20°±6.53°	0.255
Ala length & nasal bridge length index	0.16 ± 0.09	0.01 ± 0.10	0.001
Nasal tip protrusion & Nasal width Index	0.19 ± 0.12	0.22 ± 0.13	0.640
Columella length & Nasal tip protrusion Index	0.12 ± 0.79	−0.21 ± 0.16	0.220
Nasal width & ala length Index	−0.38 ± 0.31	−0.57 ± 0.45	0.251

*Values are means ± standard deviations*.

* *Indicates the two pair samples Student t-test*.

## Discussion

Tessier no. 0 cleft with a bifid nose is rare, and the etiology is still unclear. The studies have concluded the four major categories: radiation, infection, metabolic imbalances, and drugs and medicine (23). Moreover, genetics have not been well-confirmed. Some scholars have proven that genetic linkage exists and has a dominant inheritance with changeable penetrance, but the exact pattern is still unknown (7, 24). Tessier no. 0 cleft is a malformation involving a nasal supporting structure or soft tissue, manifested as enlargement or duplication of midline structures, or as agenesis or hypoplasia. The bifid nose can present a groove in the nasal columella or tip extending to a wide fissure in all nasal systems. In severe cases, a double nose may appear.

Due to the rare cases, many surgical methods are not widely accepted. Consequently, there is no consensus on a specific procedure; instead, there are many techniques based on personal experience and preference. The most significant cases were published by Ortiz Monasterio et al., who reported 59 isolated bifid noses of 176 cases of facial clefts (4). The primary method is that the dorsal nasal skin on the median line was resected, the nasal cartilage structure was reconstructed, or the bicoronal incision was used to perform lateral nasal osteotomy. However, Turkaslan et al. suggested that the incision may result in an unacceptable scar, and the bicoronal approach required further incision. They indicated that combining the intraoral and nasal approach exposed not only all nasal and lateral maxillary structures clearly to repair the nose without the previously described skin excision but also the established columellar (25). Moreover, Tawfik et al. adopted the Millard forked flap combined with external rhinoplasty in six cases and obtained good results (26). Tuersunjiang et al. also reported a case performing open rhinoplasty with an inverted-V transcolumellar incision at the middle of the columella and a Tajima incision in mild patients (27). However, there is still no standard surgical technique.

Anthropometric measurements can help surgeons evaluate the deformity objectively and quantitatively, make the deformity assessment before and after the operation, and decide the operation strategy (28). Thus, it is necessary for Tessier no. 0 cleft with a bifid nose to understand their deficiencies and shortcomings. The research by Farkas et al. proved that photogrammetry is reliable and chosen for the angular measurements (29). Thus, anthropometric methods and surgical practice intersect at one point to treat various congenital nasal deformities. Surgeons require access to face and nose databases based on accurate measurements to perform the best correction. In our study, direct anthropometric and photogrammetric methods were used to perform the angular measurements. The data of this study could be seen as a reference for plastic and maxillofacial surgeons for bifid nose evaluation and reconstructive surgery planning. Besides, these measurements could be a useful guidance for preoperative and postoperative evaluations of Tessier no. 0 cleft surgeries in the Han Chinese patients. Recently, 3D imaging systems have been widely used in facial aesthetic measurement and analysis, diagnosis of facial deformity, surgical design, and prediction, such as cleft lip and palate (7, 11, 13, 30). Ghoddousi et al. compared the accuracy of direct measurement, photogrammetric measurements, and three-dimensional body imaging technology. Results showed that the difference between the three-dimensional body imaging technology and the direct measurement method was 0.23 and 0.13 mm, respectively (31). Thus, it is a helpful tool for assessing craniofacial form, providing quantitative information about facial structures.

According to the characteristics of each patient, different surgical methods were used in our department. When more tissue sources are needed to repair the nasal coating and mucosa lining in severe patients, the expanded forehead flap is the first choice. It should be noted that the family members and patients should be fully informed before the operation. The operation involves flap transfer, flap pedicle division, and flap reversion for as long as 2 years or more. We also choose autologous costal cartilage to reconstruct the nasal supporting structure. For patients with mild symptoms, we adopted local flaps. Remarkably, the Z-shaped flap was the most widely used, which can be used to repair the local deformities of the nasal alar and tip to improve the shape of the alar and its size nostrils. For the selection of scaffold materials, nasal septum or auricular cartilage has been reported (25, 27). However, they have a small amount of tissue and are easy to absorb. Thus, we suggested not to use it, especially for patients with dysplasia of nasal cartilage.

In order to objectively understand the characteristics of the deformity and the effect of the operation, we used the applied technique of photogrammetry. Through the analysis, we could see that most items have statistical significance after surgery except the nasofrontal angle, and columella length and nasal tip protrusion index. The surgery can improve most of the problems of the patients, especially the flat and wide nasal dorsum. When comparing with the ordinary people, we could find that the nasal index and columella length, and nasal tip protrusion index of patients were relatively average due to the flat and short nose and columella deformity, but the medial canthus and nose width index was a significant difference, which showed that most patients were ocular hypertelorism and hard to improve with surgery. In males, the nasofrontal angle was significantly different, but the nasofacial angle was different in females, and the angle could return to normal after the operation. Because of nasal alar and tip defects, ala length and nasal bridge length index, nasal tip protrusion and nasal width index, and nasal width and ala length index were statistically different. Thus, these deformities can be improved by removing the excess skin and shaping the tip of the nose with cartilage. In general, Tessier no. 0 cleft with bifid nose usually has a broader nose with ocular hypertelorism and flared alae due to wider nasal width and has a non-prominent nose to shorter nasal tip protrusion and columella length.

At the same time, we also compared our two surgical methods. For the method of forehead expansion flap and costal cartilage transplantation, it could better improve the nasal facial angle by nearly 10 degrees. The index, with the tip of the nose as the apex, also improved, including ala length and nasal bridge length index, nasal tip protrusion and nasal width index, and nasal width and ala length index. It can be seen that this method is better in shaping the nasal height and more minor flaring of the alar bases. Moreover, it is better than using silica gel and local flaps to improve the angle of the ala length and nasal bridge length index, showing increased nasal tip projection, which most patients requested (2, 13), but the improvement effect of the nasolabial angle is poor. Because of the overstaffed flap at the tip of the nose and the shape of the columella is difficult to show, the angle is nearly 30° lower than that before operation. By comparison, the method of local flaps and silicone prosthesis implantation can better improve the columella length and nasal tip protrusion index due to the L-type silica gel, which could better repair the shape of the nasal columella. In general, both methods can improve the shape of the nasal tip to a certain extent, but the forehead expansion flap and costal cartilage transplantation can better improve nasal dorsum flattening and the shape of the nasal tip, and more operations are needed, while local flaps and silicone prosthesis implantation can improve the shape of the columella and need less operations and time.

At present, the optimum age of correction is a debatable issue. In the past, many scholars were against surgical intervention due to the potential damage to the development of nasal growth (32, 33). Afterward, many surgeons recommended early surgery because they found no adverse effect on nasal development (26, 34). In addition, for the adoption of costal cartilage, we usually perform surgery at the age of 6–9 years with a 60-cm chest circumference and a 120-cm body height, which has been proven to be safe and effective without affecting the chest development of the child in ear reconstruction (35). For patients where harvesting costal cartilage could not be done, we usually used silicone, which was used as a scaffold with potential tissue expansion function to further repair and adapt to facial development. At the same time, it could improve the nasal dorsum and nasal tip deformity to a certain extent. Surgeons usually recommend it and waited until the patient was mature before replacing it with autologous cartilage (36, 37). Early surgery not only corrected cosmetic defects but also avoided the continuous deterioration of deformity and psychological anxiety. Thus, we suggested that patients should be operated on early, and patients who need costal cartilage waited until complying with the requirements.

However, our study has some limitations. First, we use photogrammetric measurements to evaluate facial deformity, but its accuracy and repeatability are worse than 3D scanning. Recently, we have also started to use 3D scanning to evaluate the facial features of patients. Then errors might happen during the measurements, wherein the most common mistake is the improper identification of landmarks. For some patients with severe deformity, the landmarks of the nostrils are not clear. Also, because there was no ruler in the photographs for reference, linear measurements were not chosen in our study. Finally, this is a retrospective study that has all of the limitations of such a design.

## Conclusion

Using photogrammetric methods, it was observed that males exhibited a significantly larger nasofrontal angle, and female patients showed a relatively larger nasofacial angle. The medial canthus and nose width index, nasal tip protrusion and nasal width index, and nasal width and ala length index were both significant in males and females. For the method of forehead expansion flap and costal cartilage transplantation, it could better improve the nasal facial angle and is better in shaping the nasal height and less flaring of the alar bases. By comparison, the method of local flaps and silicone prosthesis implantation can better improve the columella length and nasal tip protrusion index. The anthropomorphic data from our study may serve as a material for nasal and reconstructive surgery.

## Data Availability Statement

The original contributions presented in the study are included in the article/Supplementary Materials, further inquiries can be directed to the corresponding author/s.

## Ethics Statement

The studies involving human participants were reviewed and approved by Plastic Surgery Hospital, Chinese Academy of Medical Sciences and Peking Union Medical College. The patients/participants provided their written informed consent to participate in this study. Written informed consent was obtained from the individual(s), and minor(s)' legal guardian/next of kin, for the publication of any potentially identifiable images or data included in this article.

## Author Contributions

XW, HW, and XZ designed and performed the experiments. XW and JY analyzed and interpreted the data. XW searched the literature and wrote the manuscript. FF revised the manuscript. All authors contributed to the article and approved the submitted version.

## Funding

This study was funded by The Foundation of the Chinese Academy of Medical Sciences—Plastic Surgery Hospital (3060120043).

## Conflict of Interest

The authors declare that the research was conducted in the absence of any commercial or financial relationships that could be construed as a potential conflict of interest.

## Publisher's Note

All claims expressed in this article are solely those of the authors and do not necessarily represent those of their affiliated organizations, or those of the publisher, the editors and the reviewers. Any product that may be evaluated in this article, or claim that may be made by its manufacturer, is not guaranteed or endorsed by the publisher.

## Supplementary Material

Supplementary Figure 1 Computed tomography showed the nasal structure of the patients in Figure 2. No defects and deformities were found in the nasal bone.
