# Supplementary figures and images for: Morphological Analysis of Nose in Patients of Tessier No. 0 Cleft With a Bifid Nose in China
Source: Front Pediatr. 2021 Nov 29;9:768176. doi: 10.3389/fped.2021.768176 (PMC8668193; doi:10.3389/fped.2021.768176)

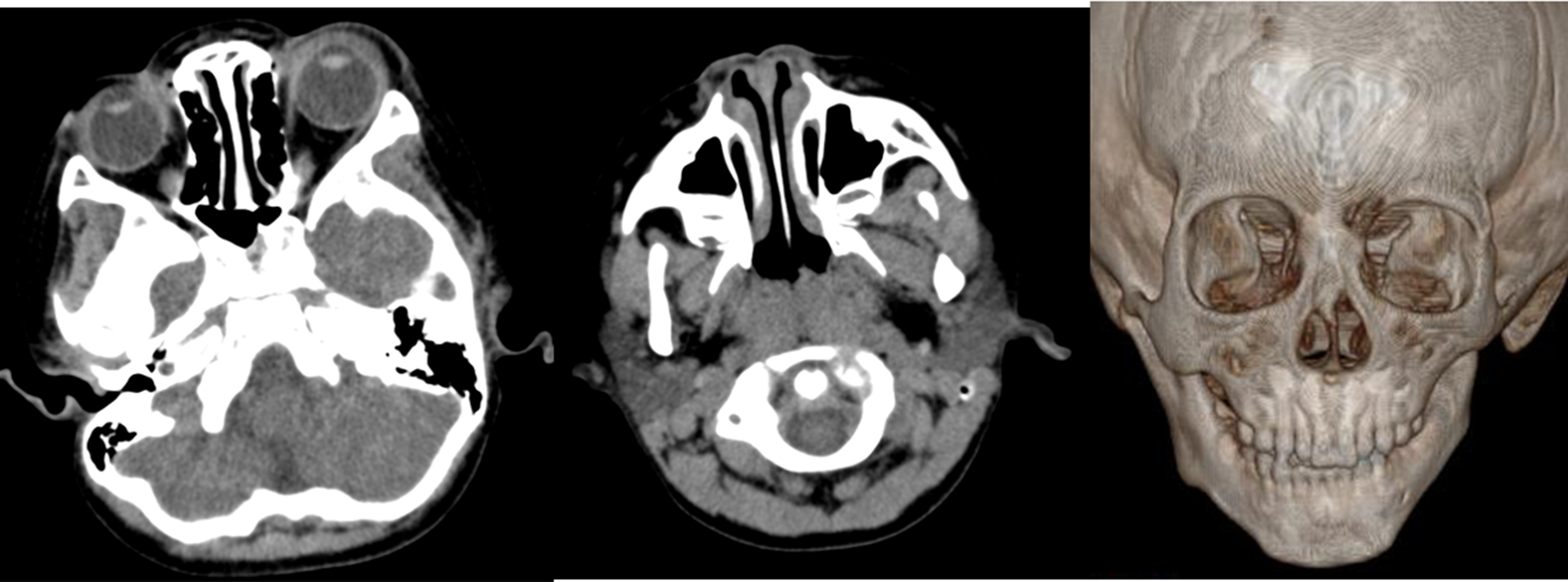

Supplement: Supplementary Figure 1 — Computed tomography showed the nasal structure of the patients in Figure 2. No defects and deformities were found in the nasal bone. [file Image_1.TIF]
